# Full-length transcriptome, proteomics and metabolite analysis reveal candidate genes involved triterpenoid saponin biosynthesis in *Dipsacus asperoides*

**DOI:** 10.3389/fpls.2023.1134352

**Published:** 2023-02-10

**Authors:** Jie Pan, Chaokang Huang, Weilin Yao, Tengfei Niu, Xiaolin Yang, Rufeng Wang

**Affiliations:** ^1^ Institute of Chinese Materia Medica, Shanghai University of Traditional Chinese Medicine, Shanghai, China; ^2^ The SATCM Key Laboratory for New Resources and Quality Evaluation of Chinese Medicines, Shanghai University of Traditional Chinese Medicine, Shanghai, China; ^3^ Shanghai R&D Center for Standardization of Chinese Medicines, Shanghai, China; ^4^ The MOE Key Laboratory for Standardization of Chinese Medicines, Shanghai University of Traditional Chinese Medicine, Shanghai, China

**Keywords:** *Dipsacus asperoides*, saponin distribution, biosynthesis, transcriptome, proteomics

## Abstract

*Dipsacus asperoides* is a traditional medicinal herb widely used in inflammation and fracture in Asia. Triterpenoid saponins from *D. asperoides* are the main composition with pharmacological activity. However, the biosynthesis pathway of triterpenoid saponins has not been completely resolved in *D. asperoides*. Here, the types and contents of triterpenoid saponins were discovered with different distributions in five tissues (root, leaf, flower, stem, and fibrous root tissue) from *D. asperoides* by UPLC-Q-TOF-MS analysis. The discrepancy between five tissues in *D. asperoides* at the transcriptional level was studied by combining single-molecule real-time sequencing and next- generation sequencing. Meanwhile, key genes involved in the biosynthesis of saponin were further verified by proteomics. In MEP and MVA pathways, 48 differentially expressed genes were identified through co-expression analysis of transcriptome and saponin contents, including two isopentenyl pyrophosphate isomerase and two 2,3-oxidosqualene β-amyrin cyclase, etc. In the analysis of WGCNA, 6 cytochrome P450s and 24 UDP- glycosyltransferases related to the biosynthesis of triterpenoid saponins were discovered with high transcriptome expression. This study will provide profound insights to demonstrate essential genes in the biosynthesis pathway of saponins in *D. asperoides* and support for the biosynthetic of natural active ingredients in the future.

## 1 Introduction

*Dipsacus asperoides* belonging to the Dipsacaceae family is a kind of widely applied traditional Chinese medicinal crops (Wan et al., 2021). The dried root of *D. asperoides* known as “Xu Duan” is frequently prescribed for the treatments of fracture and impotence due to its beneficial health properties. Over the last decade, the wild resource of *D. asperoides* was over-exploited and the demand for this medicinal plant has been progressively increasing (Wang et al., 2016). Therefore, the researches of botany, cultivation, molecular biology, and metabolic engineering in *D. asperoides* are indispensable for the effective production of bioactive secondary metabolites in natural medicinal plants or crops, which predominantly count on the elucidation of biosynthesis pathway in these secondary metabolites. Up to now, large amounts of research has been conducted and evaluated on chemical compositions (Yu et al., 2019) and pharmacological (Yu et al., 2012) activities of *D. asperoides*. Modern pharmacological research has verified that the saponin extract of *D. asperoides* had numerous significant biological activities, such as anti-inflammatory (Li et al., 2013; Lu et al., 2020), anti-oxidatant (Tran et al., 2008), Alzheimer’s disease inhibitory (Ji et al., 2012; Yu et al., 2012; Wang et al., 2018), antifungal (Choi et al., 2017), anti-apoptotic (Lu et al., 2020), and anti-cancer (Jeong et al., 2008), etc. The studies of chemical analysis and isolation on *D. asperoides* showed that its chemical compositions mainly consisted of triterpenoid saponins (Jung et al., 1993), iridoid glycosides (Sun et al., 2015) and alkaloids (Li et al., 2013), etc. Triterpenoid saponins including asperosaponin VI, hederagenin and alpha-Hederin are the principal bioactive components of *D. asperoides* (Liu et al., 2011; Wang et al., 2020). Previous research showed that the content of asperosaponin VI was dissimilar in different tissues of *D. asperoides*, as well as in various habitats (Jin et al., 2020). Nevertheless, the content distributions of saponins in different tissues of *D. asperoides* have not been investigated.

Since triterpenoid saponins are the principal active components in *D. asperoides*, it is vital for revealing candidate genes involved in the biosynthetic pathways of triterpenoid saponins. Saponins are originally derived from isopentenyl diphosphate (IPP) in the cytosol mevalonic acid (MVA) pathway and plastid methylerythritol phosphate (MEP) pathway (Thimmappa et al., 2014). Two molecules of IPP and one molecule of dimethylallyl diphosphate (DMAPP) are catalyzed to form farnesyl pyrophosphate (FPP) through geranyl pyrophosphate synthase (GPS) and farnesyl pyrophosphate synthase (FPS) (Vranova et al., 2013). Then 2,3-oxidosqualene is derived from two molecules of FPP *via* squalene synthase (SS) and squalene epoxidase (SE), whereafter diverse oxidosqualene cyclase (OSC) enzymes catalyze 2,3-oxidosqualene to a series of triterpene backbones, such as β-amyrin, dammarane and phytosterol (Cheng et al., 2020). β-Amyrin and other products are further oxidated and hydroxylated by cytochromep450 (CYPs) monooxygenases and glycosylated *via* UDP- glycosyltransferases (UGTs) at the C-3 or C-28 positions to generate various triterpenoid saponins (Seki et al., 2015). Recently, researches have been certified the pivotal function of different enzymes in the synthesis of the triterpene skeleton (Wang Y. et al., 2022; Wang Z. L. et al., 2022). However, the genes related to the modification of saponins in *D. asperoides* remain to be comprehensively illuminated.

Currently, metabolomics and transcriptomics have been extensively performed to clarify the correlation of components and key genes involving saponin biosynthesis. Saponins as paramount pharmacological chemicals have various distribution patterns in medicinal plants of different tissues (Jia et al., 2013). In this study, ultra-performance liquid chromatography-quadrupole time-of-fight mass spectrometry (UPLC-Q-TOF-MS) was applied to explore the contents of triterpenoid saponins and distribution patterns of saponin in five different tissues from *D. asperoides*, including roots, leaves, flowers, stem, and fibrous roots. Meanwhile, single-molecule real-time (SMRT) sequencing and next-generation sequencing (NGS) techniques were jointly used to obtain an outright transcriptome dataset of *D. asperoides*. By analyzing the relationship of different triterpenoid saponins and sequencing data in five tissues, some tissue-specific patterns of specific genes and saponins were discovered in *D. asperoides*. Then the weighted gene co-expression network analysis (WGCNA) (Langfelder and Horvath, 2008) was further applied to identify critical hub genes attached to the biosynthesis of triterpenoid saponins. Moreover, proteomics technology was used to study the discrepancies in protein levels of three *D. asperoides* tissues comprising roots, leaves, and flowers. Finally, the candidate genes involved triterpenoid saponin biosynthesis in *D. asperoides* were revealed by multiple omics strategy. This study will provide profound insights to get essential genes in saponin biosynthesis pathway and lay a foundation for biosynthetic natural ingredients in *D. asperoides*.

## 2 Materials and methods

### 2.1 Plant materials

The fresh samples of *D. asperoides* were collected from Baoshan, Yunnan, China (25°06′43″N, 99°09′42″E). The fresh specimens were carefully cleaned and immediately separated into five tissues (root, leaf, flower, stem, and fibrous root) to store for the following experiments.

### 2.2 Chemical compositional analysis

#### 2.2.1 Sample preparation

Each tissue sample was dried in an oven at 50°C, and 500 mg powder of each sample was added to 25 mL of 70% methanol. Ultrasonication was conducted for 1 h at room temperature (100 W, 40 kHz). Then, all prepared samples were centrifuged at 14,000 rpm for 30 min, and corresponding supernatants were used for analysis by UPLC-Q-TOF-MS.

#### 2.2.2 Standard preparation

Standards (loganin, sweroside, loganic acid, hederagenin, alpha-Hederin, dipsacoside B, asperosaponin VI and hederacoside C) were purchased from Chengdu MUST Biotechnology Co (Chengdu, China). The purity of each standard substance was above 98%. Pre-weighed standards were dissolved in methanol at the final concentration of 0.1 mg/mL, and all standard solutions were stored at 4°C.

#### 2.2.3 UPLC-Q-TOF-MS analysis

The contents of chemicals were determined as described in the literature (Tao et al., 2019) with minor modifications. The analytical facility contained an UPLC system (Shimadzu, Japan) and a Q-TOF 5600^+^ mass spectrometer provided with Turbo V sources (AB sciex, USA). The chromatographic conditions were set as below: Waters ACQUITY UPLC HSS T3 (2.1 mm × 100 mm, 1.8 μm); sample injection volume, 5 μL; temperature of column oven, 35°C; flowrate, 0.4 mL/min; mobile phases, water with 0.1% formic acid (solvent A) and acetonitrile (solvent B). A gradient programmer was employed as follows: 5% B (0 - 2 min), 5-30% B (2.0 - 8.0 min), 30-45% B (8.0 - 9.0 min), 45-60% B (9.0 - 10.0 min), 60-80% B (10.0 - 16.0 min), 80-95% B (16.0 - 21.0 min), 95-100% B (21.0 - 22.0 min). The operating parameters for Q-TOF-MS were set as below: full-scan data acquisition was performed from m/z 100 to 1,500 in the negative mode; ion spray voltage, - 4.5 kV; collision energy, - 35 eV.

### 2.3 Transcriptomic analysis

#### 2.3.1 RNA preparation, illumina library preparation and sequencing

The tissues (root, leaf, flower, stem, and fibrous root) of *D. asperoides* were used for illumina library preparation and sequencing. In brief, the total RNA was extracted from each tissue using TRIzol^®^ Reagent (Magen). Each total RNA sample was then used for NGS analysis, while equivalent amounts of RNA from roots, leaves, flowers, stems, and fibrous roots were mixed for SMRT analysis.

The first-strand cDNAs were synthesized with random hexamer primers and Reverse Transcriptase (RNase H) using mRNA fragments as templates, followed by second-strand cDNA synthesis using DNA polymerase I, RNAseH, buffer, and dNTPs. Adaptor-ligated cDNA was used for PCR amplification. PCR products were purified (AMPure XP system) and library quality was assessed on an Agilent Bioanalyzer 4150 system. Finally, sequencing was performed with an Illumina Novaseq 6000/MGISEQ-T7 instrument. Raw data obtained from the transcriptome sequencing by removing the adapter sequence and filtering out low-quality reads to gain high-quality clean reads was used for subsequent analysis. Clean data were used to do *de novo* assembly with Trinity. The assembled transcriptome sequences were compared with five databases (NR, SwissProt, Pfam, GO and KEGG databases) to obtain the annotation information in each database.

#### 2.3.2 SMRT library construction, sequencing, and data analysis

The RNA extracted from five tissue types was mixed into one specimen to establish SMRT library. Full-length cDNA was produced using a SMARTer PCR cDNA Synthesis Kit (Clontech), and isoform sequencing (Iso-Seq) libraries were constructed using a SMRTbell™ Template Prep Kit 1.0 (Pacific Biosciences, Menlo Park, CA, USA). Sequencing was performed on a PacBio Sequel II instrument with a Sequel™ Sequencing Kit 2.0 (Pacific Biosciences). Functional annotations were conducted using BLAST (version 2.2.26) against different protein and nucleotide databases including the NR database, Swissprot database, Gene Ontology (GO) database, eggNOG (Evolutionary Genealogy of Genes: Non-super-vised Orthologous Groups) database, and KEGG (Kyoto Encyclopedia of Genes and Genomics) database. Principal component analysis (PCA) is an important analytical method that analyzes the multiple sets of data and interprets it with fewer principal components, while visualizing differences and interpreting most characteristics of the original data (Wang et al., 2012). Heatmap was plotted by an online platform for data analysis and visualization (https://www.bioinformatics.com.cn).

### 2.4 Label-free proteomic analysis

#### 2.4.1 Protein extraction and LC-MS/MS analysis

SDT (4% SDS, 100 mM Tris-HCl, 1 mM DTT, pH 7.6) buffer was used for sample analysis and protein extraction. The amount of protein was quantified with the BCA Protein Assay Kit (Bio-Rad, USA). Protein digestion was performed according to filter-aided sample preparation (FASP) procedure described by Matthias Mann. The digest peptides of each sample were desalted on C18 Cartridges (Empore™ SPE Cartridges C18 (standard density), bed I.D. 7 mm, volume 3 mL, Sigma), concentrated by vacuum centrifugation and reconstituted in 40 µl of 0.1% (v/v) formic acid. The proteins were separated on 12.5% SDS-PAGE gel (constant current 14 mA, 90 min). LC-MS/MS analysis was performed on a Q Exactive mass spectrometer (Thermo Scientific) that was coupled to Easy nLC (Thermo Fisher Scientific) for 120 min. The peptides of each sample were re-separated using a reverse phase trap column (Thermo Scientific Acclaim PepMap 100, 100 μm × 2 cm, nanoViper C18), with the C18-reversed phase analytical column in buffer A (0.1% Formic acid) and separated with a linear gradient of buffer B (84% acetonitrile and 0.1% formic acid) at a flow rate of 300 μL/min controlled by IntelliFlow technology. The mass spectrometer was operated in positive ion mode and the data was determined as described in the literature (Chen et al., 2020).

#### 2.4.2 Protein identification, quantification and bioinformatic analysis

The MS raw data for each sample were combined and searched using the Max Quant 1.5.3.17 software for identification and quantitation analysis (Chen et al., 2020). The transcriptome of *D. asperoides* database was used for protein identification, and the database pattern was reversed. The protein sequences of the selected differentially expressed proteins were locally searched using the NCBI BLAST+ client software and InterProScan to find homologue sequences, then terms were mapped and sequences were annotated using Blast2GO. The GO annotation results were plotted by R scripts. Following annotation steps, proteins were blasted against KEGG database to retrieve orthology identifications and were subsequently mapped to pathways. Enrichment analysis was applied based on the Fisher’ exact test, considering the whole quantified proteins as the background dataset. Benjamini-Hochberg correction for multiple testing was further applied to adjust derived *p*-values. And only functional categories and pathways with *p*-values under a threshold of 0.05 were considered significant. Data are available *via* ProteomeXchange with identifier PXD038580.

### 2.5 Gene co-expression network analysis

The WGCNA V1.41-1 R package was applied to conduct co-expression and module analyses (Langfelder and Horvath, 2008).

## 3 Results and discussion

### 3.1 Relative quantification assessment of differential compounds in *D. asperoides*

**Figure 1:**
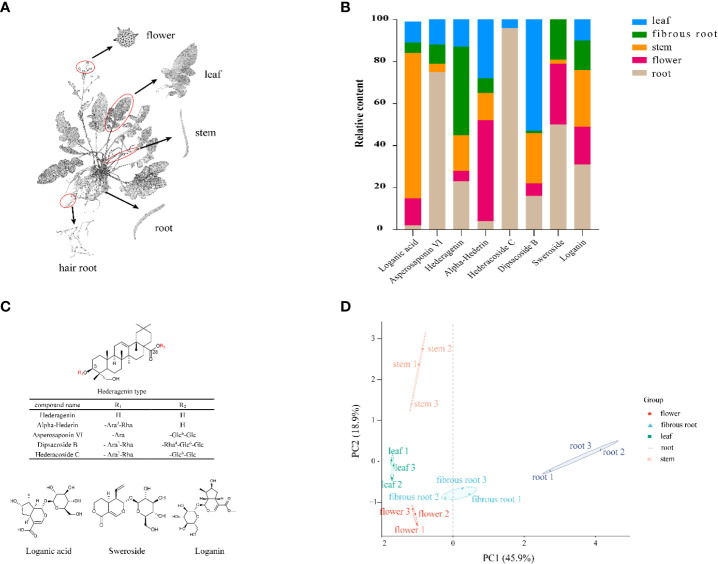
Structural, chemometric analyses and compositional variations in five tissues by UPLC-Q-TOF-MS in *D. asperoides*. **(A)** Five tissues of *D. asperoides* were analyzed in this study: root, leaf, flower, stem, and fibrous root. **(B)** Compositional variations in five tissues of *D. asperoides*. **(C)** Chemical structures of compounds isolated from *D. asperoides*. Glc, glucopyranosyl; Ara, arabinopyranosyl; Rha rhamnopyranosyl. **(D)** PCA analysis of relative quantification of differential compounds in five tissues.

*D. asperoides* is a perennial plant commonly used as a traditional Chinese medicinal crop and mainly grows in the southern regions of China, such as Yunnan and Hunan Provinces (Yu et al., 2019). *D. asperoides* has been testified with pharmacological benefits for the treatments of a wide range of diseases, such as anti-inflammatory, anti-oxidatant, analgesic and anti-osteoporosis (Hung et al., 2006). To disclose the chemical compositions of *D. asperoides*, UPLC-Q-TOF-MS was used to investigate the distinct metabolites in aerial (leaves, flowers, and stems) and underground sections (roots and fibrous roots) (**Figure 1A**). As expected, eight components exhibit significant differences among these tissues (**Figure 1B**), including loganin, sweroside, loganic acid, hederagenin, alpha-Hederin, dipsacoside B, asperosaponin VI and hederacoside C (**Figure 1C**). Saponins presented great contents in the root of *D. asperoides*, including but not limited to hederacoside C and asperosaponin VI. Hederagenin was more abundant in fibrous roots than in other tissues, and asperosaponin VI was more abundant in roots. Hederacoside C was only detected in roots and leaves. Alpha-hederin and dipsacoside B were highly abundant in flowers and leaves, respectively. Notably, alpha-hederin, dipsacoside B and hederagenin have huge contents in flowers, leaves, fibrous roots, respectively. The study also discovered some non-saponins such as loganin and sweroside presented high contents in the root of *D. asperoides* compared to other tissues, but loganic acid was more abundant in stems. Through principal component analysis, it was found that the significant differences between root and stem tissues and other tissues (**Figure 1D**). However, only little differences were shown among fibrous root, flower and leave tissues. The analysis indicated that the relative content of compounds in the root was significantly different from that in the flower, which would be conducive to the further analysis of the relationship between the different compounds and differentially genes in the two tissues. The above results indicated the structure-specific and tissue-specific dependent patterns of saponins in *D. asperoides*.

### 3.2 Transcriptomic analysis and annotation

It is more accessible to explore metabolic processes of triterpenoid saponins in plants through the analysis of changes in compounds combined with functional genetics. NGS is capable of sequencing dozens or millions of DNA molecules synchronously and is used to analyze transcriptomes to get quantitative levels of gene expression. Nevertheless, the sequencing quality is relevant to the reading length and the synergy of gene cluster replication (Xu et al., 2020). SMRT sequencing can avoid the limitations of short-read sequences to obtain more long read length (Zhong et al., 2020). In this work, NGS and SMRT techniques were combinedly used to precisely assemble a comprehensive transcriptome of *D. asperoides*. The full-length transcriptome of *D. asperoides* was obtained using PacBio SMRT sequencing. The SMRT sequencing and next-generation sequencing (NGS) were concurrently combined to get more accurate transcriptomic database. First, all RNA specimens were sequenced by Illumina Novaseq 6000/MGISEQ-T7 instrument, generating 97.45 GB clean reads and Q30 up to 92.33% (**Supplementary Table 1**). Subsequently, 460,177 reads of insert were gained by SMRT sequencing, comprising a total of 420,803 full-length non-chimeric reads that incorporated 5’/3’-primers and a poly-(A) tail, along with 38,200 non-full length reads. To get high-quality isoforms with accuracy greater than 99%, iterative clustering for error correction was applied for predicting consensus isoforms, where the redundant sequences were clustered together to obtain a new consistency sequence, and then the non-full-length sequences are compared with the consistency sequence by quiver program. In total, 47,323 consensus isoforms were obtained, including 47246 high-quality (HQ) and 77 low-quality (LQ) transcripts. The clean Illumina reads were used to correct all SMRT reads to reduce high subread error rates, and the CD-HIT software was used to cluster the redundant sequences, obtaining 19526 unigene, with a mean length of 1961 bp, N50 of 2180 bp and GC content of 41%. To obtain a full-scale annotation of *D. asperoides* transcriptome, all full-length transcripts were annotated through NR, GO, SwissProt, KEGG, and Pfam databases (**Supplementary Figure 1**). Based on GO annotation, transcripts were sorted into the biological processes (BP), cellular component (CC), and molecular function (MF) (**Supplementary Figure 2**). A total number of 10383 transcripts were annotated in the KEGG database and classified into five main categories as follows: cellular processes (1069), environmental information processing (1023), genetic information processing (2104), metabolism (4436) and organismal Systems (1751) (**Supplementary Figure 2**). Notably, the “metabolic” pathways include the metabolism of terpenoids and polyketides (208), biosynthesis of other secondary metabolites (232) and carbohydrate metabolism (916). Furthermore, in the GO and KEGG enrichment analysis of differentially upregulated genes in roots and flowers (**Supplementary Figure 3**), it was shown that 486 transcripts were found in the “biosynthesis of secondary metabolites”, which would contribute to revealing the biosynthesis pathways of saponins in *D. asperoides* in the future. The transcriptome data were deposited in NCBI with accession number PRJNA889678. The high-quality full-length transcriptome of *D. asperoides* offers much more information to reveal candidate genes involved triterpenoid saponins biosynthesis than other reports.

### 3.3 Tissue-specific dependent patterns of saponin-related genes in *D. asperoides*

**Figure 2:**
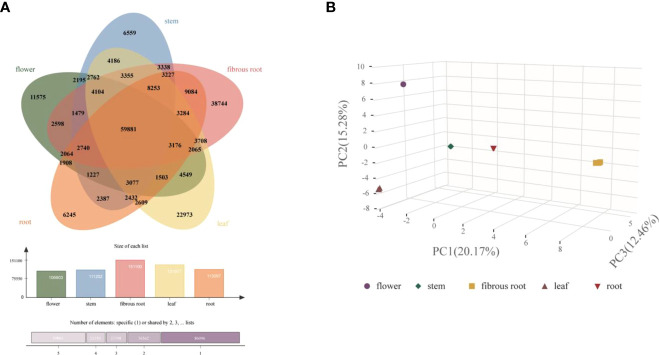
Clustering analysis of gene expression in five tissues of *D. asperoides*. **(A)** A Venn diagram comparing gene expression between different tissues. **(B)** PCA of gene expression in five tissues.

**Figure 3:**
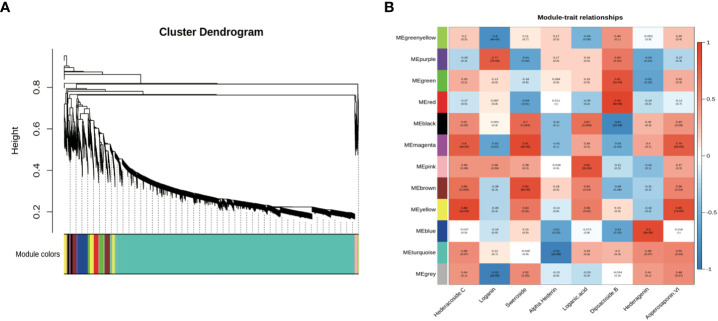
Co-expression network analysis of genes related to different saponins in five tissues of *D. asperoides* (root, leaf, flower, stem, fibrous root). **(A)** Gene dendrogram obtained by average linkage hierarchical clustering, demonstrating co-expression modules in *D. asperoides*. **(B)** The correlation coefficient between modules and saponins in *D. asperoides*.

To integrally analyze gene expression patterns in different *D. asperoides* tissue samples, PCA and Venn diagrams were established by processing transcriptome data. It was shown that there were significant differences among these five tissue samples (**Figure 2B**) with PC1, PC2, and PC3 interpretations varied by 20.17%, 15.28%, and 12.46%, respectively. In Venn diagram, 59881 transcripts were expressed in all five tissues, and 6245, 11575, 6559, 38744, and 22973 transcripts were particularly expressed in roots, flowers, stems, fibrous roots, and leaves, respectively (**Figure 2A**). Differentially expressed genes (DEGs) were further identified by comparing gene expression levels among samples, using coefficients calculated from log2 (fold change) and *p*-values for each transcript. Finally, 3575, 3696, and 4596 DEGs were found by comparing roots, stems and fibrous roots to flowers, respectively (**Table 1**). Different specific variations of triterpenoid saponins in *D. asperoides* are related to the expression of biosynthetic key genes. In MVA and MEP pathways, 2,3-oxidosqualene is the precursor to biosynthesis of triterpenoid saponins (**Figure 3**). SS and SE were responsible for the critical step of terpenoid carbocyclic skeleton compounds and intermediates biosynthesis (Xu et al., 2004). In addition, CYPs and UGTs were both significant to the diversification of triterpenoid saponin structures (Cheng et al., 2020). Beta-amyrin cyclase (β-AS) can cyclize 2,3-oxidosqualene to form β-amyrin. β-amyrin can be catalyzed to hederagenin through two CYPs, and hederagenin was further glycosylated to generate diverse saponins by UGTs. It was reported that CYP716A94 as a β-amyrin 28-oxidase could catalyze β-amyrin to oleanolic acid and CYP72A68 was essential to produce hederagenin through hydroxylation of C-23 in oleanolic acid (Han et al., 2018; Tzin et al., 2019). UGT71G1 and UGT73K1 could catalyze the glycosylation of C-28 or C-3 hydroxyl group in hederagenin to produce hederagenin 3-*O*-glucoside or 28-*O*-glucoside (Achnine et al., 2005). Up to now, only a few CYPs and UGTs in *D. asperoides* have been functionally identified.

**Table 1:** The up-regulated and down-regulated DEGs in different tissues of *D. asperoides* (*p*-values ≤ 0.05 and fold change ≥ 2).

Item	Up-Regulated	Down-Regulated	Total
root -VS- flower.DEseq2	1365	2210	3575
root -VS- stem.DEseq2	548	831	1379
root -VS- fibrous root.DEseq2	395	1325	1720
root -VS- leaf.DEseq2	1117	1236	2353
stem -VS- flower.DEseq2	1547	2149	3696
stem -VS- fibrous root.DEseq2	511	964	1475
stem -VS- leaf.DEseq2	631	1660	2291
leaf -VS- flower.DEseq2	1319	2008	3327
leaf -VS- fibrous root.DEseq2	877	1393	2270
flower -VS- fibrous root.DEseq2	2297	2299	4596

### 3.4 Identification of transcripts potentially involved in saponin biosynthesis

**Figure 4:**
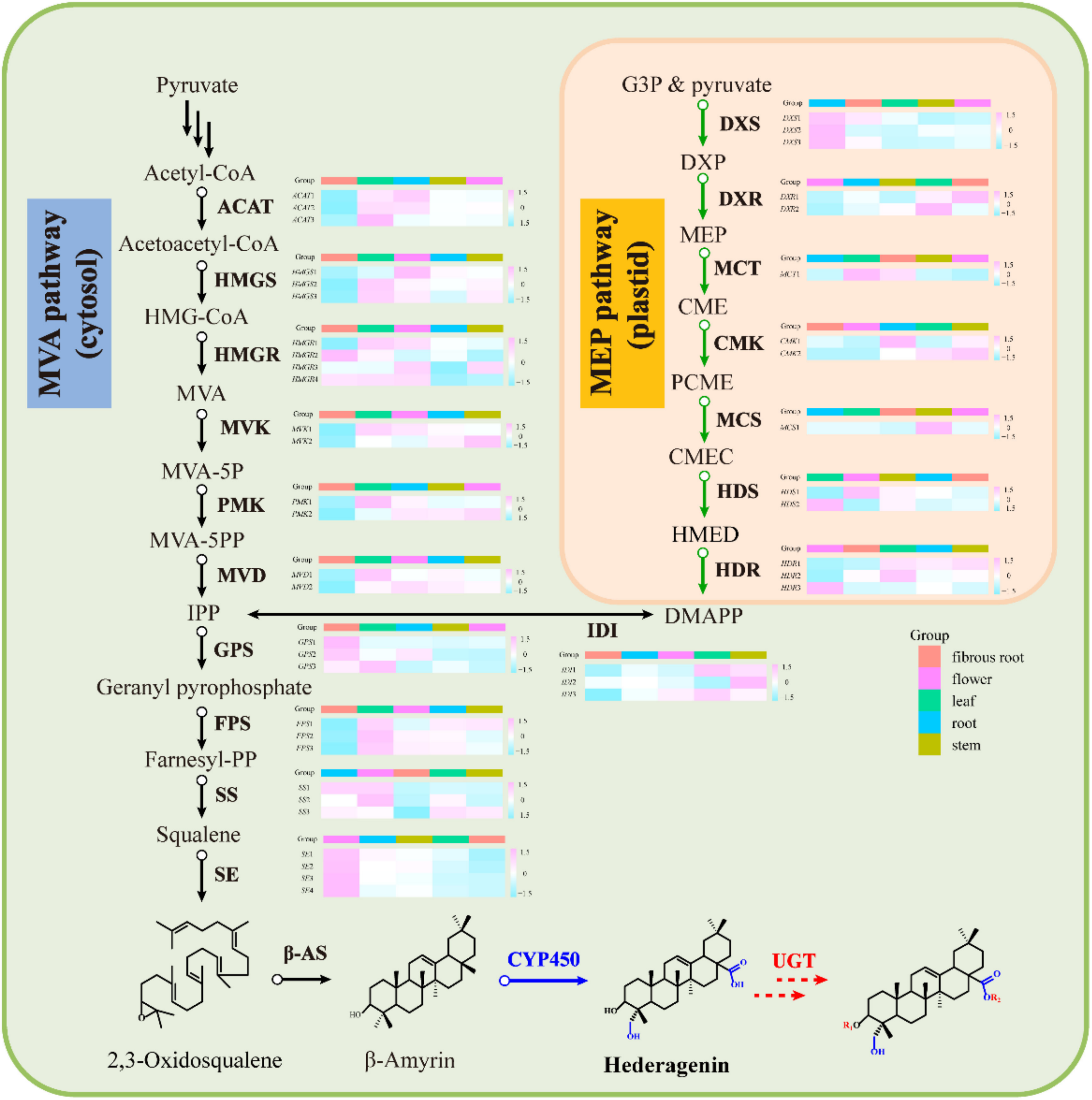
Gene expression in the MVA and MEP pathway for saponins in *D. asperoides*. ACAT, Acetyl Coenzyme a Acyltransferase; HMGS, 3-Hydroxy-3-Methylglutaryl Coenzyme a Synthase; HMGR, Hydroxymethylglutaryl-Coa Reductase; MVK, Mevalonate Kinase; PMK, Phosphomevalonate Kinase; MVD, Mevalonate Pyrophosphate Decarboxylase; IDI, Isopentenyl Diphosphate Delta-Isomerase; GPS, Geranyl Pyrophosphate Synthase; FPS, Farnesyl Pyrophosphate Synthase; SS, Squalene Synthase; SE, Squalene Epoxidase; DXS, 1-Deoxy-D-Xylulose-5-Phosphate Synthase; DXR, 1-Deoxy-D-Xylulose-5-Phosphate Reductoisomerase; MCT, 2-C-Methyl-D-Erythritol 4-Phosphate Cytidylyltransferase; CMK, 4-(cytidine 50-diphospho)-2-C-methyl-D-erythritol kinase; MCS, 2-C-Methyl-D-Erythritol 2,4-Cyclodiphosphate Synthase; HDS, 4-Hydroxy-3-Methylbut-2-En-1-Yl Diphosphate Synthase; HDR, 1-Hydroxy-2-Methyl-2-(E)-Butenyl 4-Diphosphate Reductase; HMG-CoA, 3-Hydroxy-3-Methylglutaryl CoA; DXP, 1-Deoxy-D-Xylulose 5-Phosphate; CMEC, Carboxymethyl Ethyl Cellulose; DMAPP, Dimethylallyl Diphosphate; MVA-5P, Mevalonate-5-Pyrophosphate; CME, 4-(Cytidine 5’-Diphospho)-2-C-Methyl-D-Erythritol; PCME, 2-Phospho-4-(Cytidine 5’-Diphospho)-2-C-Methyl-D-Erythritol; HMED, 4-Hydroxy-3-Methylbut-2-Enyl-Diphosphate; β-AS, Beta-Amyrin Cyclase; CYP450, Cytochromep450; UGT, Glycosyltransferase; MVA, Mevalonic acid; IPP, Isopentenyl Diphosphate; MVA, Schematic of Mevalonate; MEP, 2-C-Methyl-D-Erythritol 4-Phosphate/1-Deoxy-D-Xylulose 5-Phosphate.

Transcripts related to upstream or downstream genes of saponin biosynthesis were found by analyzing the transcriptome of five tissue samples (roots, leaves, flowers, stems, fibrous roots). As shown in PCA (**Figure 2B**), the flower group was more differentiated from other groups, while the root group was clustered at the center. A total of 48 DEGs involved in MEP and MVA pathways were identified (**Supplementary Table 2**), and 4 (8.33%), 6 (12.50%), 7 (14.59%), 13 (27.08%), and 18 (37.50%) genes had prominent expression levels in flowers, roots, stems, fibrous roots, and leaves tissues, respectively. For instance, transcript-39509 and transcript-40019 (isopentenyl pyrophosphate isomerases, IDI) were expressed at higher levels in the leaf than in other tissues. Transcript-8753 (1-deoxy-D-xylulose-5-phosphate synthase, DXS) and transcript-23667 (GPS) showed higher expression levels in root and flower than other tissues, respectively. Transcript-9193 (hydroxymethylglutaryl-CoA reductase, HMGR), transcript-31058 (mevalonate kinase, MVK), transcript-31919 (4-diphosphocytidyl-2-C-methyl-D-erythritol kinase, CMK) and TR4556_c1_g2 (2-C-methyl-D-erythritol 2,4-cyclodiphosphate synthase, MCS) were supremely expressed in stem tissues. Transcript-18826 (hydroxymethylglutaryl-CoA synthase, HMGS), transcript-25812 (4-hydroxy-3-methylbut-2-enyl diphosphate reductase, HDR) and transcript-25516 (SS) showed high expression levels in fibrous root tissues. Transcript-14566, transcript-16847, transcript-5418 and transcript-10764 as SE also showed high expression levels in fibrous root tissues, as well as transcript-7961 and transcript-7223 (β-AS). In leaves tissues, two acetoacetyl-CoA thiolases (transcript-23660 and transcript-24461), three farnesyl diphosphate synthases (transcript-28558, transcript-24094 and transcript-28255), phosphomevalonate kinase (transcript-17266) and mevalonate pyrophosphate decarboxylase (transcript-25465) had remarkably expression levels (**Figure 4** and **Supplementary Table 2**). Through hierarchical clustering analysis and gene expression modes in different tissues, upstream genes were identified that potentially participated in the triterpene saponin biosynthesis of *D. asperoides*.

Furthermore, CYPs and UGTs that related to the downstream biosynthetic pathway of triterpenoid saponin were screened in *D. asperoides* transcriptome. All 125 CYP transcripts were discovered, of which 13, 15, 23, 35 and 39 (10.4%, 12.0%, 18.4%, 28.0%, 31.2%) had the highest expression in fibrous root, stem, root, leaf, and flower (**Supplementary Figure 4 and Supplementary Table 3**). Meanwhile, 230 UGTs were identified, of which 29, 29, 33, 50 and 89 (12.61%, 12.61%, 14.35%, 21.74%, 38.69%) had the highest expression in flower, stem, leaf, fibrous root, and root (**Supplementary Figure 4 and Supplementary Table 3**). According to the above results, a presumable conclusion could be drawn that the expression of CYPs and UGTs was different in five tissues, leading to differential contents of triterpenoid saponins.

### 3.5 Proteomics bioinformatics analysis

**Figure 5:**
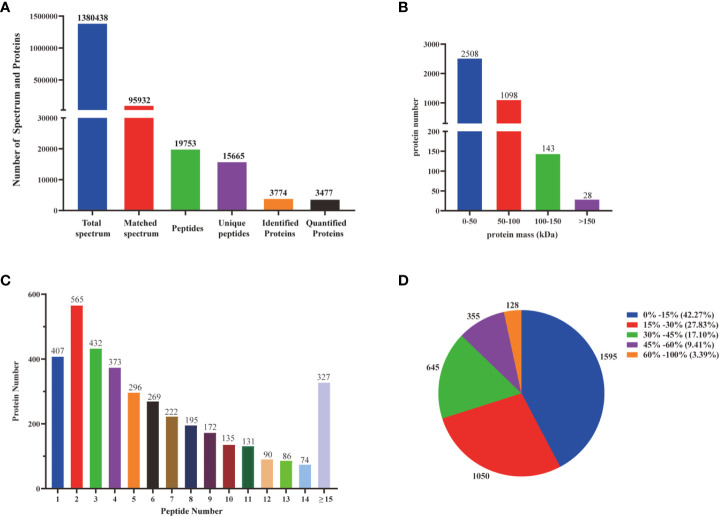
Identification and analysis of the proteome on *D. asperoides*. **(A)** Total spectrum, matched spectrum, peptides, unique peptides, identified proteins, and quantified proteins detected from Label- free proteomic analysis. **(B)** Identified proteins were grouped based on their protein mass. **(C)** The number of peptides matched to proteins was shown by Protein Pilot 5.0. **(D)** The identified proteins were classified into pie charts by protein sequence coverage.

Transcriptomic analysis can only reveal triterpenoid saponin biosynthesis at the mRNA level, but cannot explain post-transcriptional processes such as translation and protein modification. Proteins are considered to have a greatly direct correlation with triterpenoid saponin. In this study, Label-free quantitative LC-MS/MS was used to obtain a full-scale proteomic profiles of three *D. asperoides* tissues. A total of 1,380,438 spectrums, 95,932 matched spectrums, 15,665 unique peptides and 3,774 identified proteins (**Figure 5A**) were collected. There were 2508, 1098, 143, and 28 proteins with molecular weights of 0-50 kDa, 50-100 kDa, 100-150 kDa, and over 150 kDa (**Figure 5B**), respectively. The above proteins with 1-5 peptides, 6-10 peptides, 11-14 peptides, and 15 or more peptides consisted of 2073, 993, 381 and 327 (**Figure 5C**), respectively. Protein sequences converging with 0-15%, 15-30%, 30-45%, 45-60% and 60-100% scope were accounted for 42.27%, 27.83%, 17.10%, 9.41%, and 3.39% (**Figure 5D**), respectively. As shown in **Supplementary Figure 5**, 643 out of 3,735 proteins were expressed in all three tissues, whereas 84, 103, and 475 were exclusively expressed in root, leaf, and flower, indicating that there were distinct proteins in different tissues. Therefore, significant differences in proteins were detected by comparing protein expression profiles between tissues using the fold change (FC) ≥ 2 and *p*-values < 0.05. Comparing Pleaf and Pflower samples with Proot samples, 102 and 132 proteins were discovered, respectively. Meanwhile, 740 differentially proteins were identified in Pleaf and Pflower samples (**Supplementary Table 5**). Proteomics analysis was further conducted to examine the changes in different tissues from protein levels as verified supplementary for transcriptome. In this study, it was found that there were significant differences between root and flower tissues by the analysis of compounds in five tissues (**Figure 1D**). Hence, the GO and KEGG enrichment analysis of different proteins in root and flower tissues were conducted. After go analysis, the above peptides were divided into BP (2985), MF (2266) and CC (3833). KEGG analysis showed that these different proteins were further assigned into 194 biological pathways, such as biosynthesis of cofactors, proteasome, glycerolipid metabolism, etc. (**Supplementary Figure 6**). In addition, heatmaps for all proteins were performed between three tissues. As shown in **Supplementary Figure 7**, the differentially proteins between various groups were diverse (**Supplementary Table 6**). Compared with the study on the proteomic analysis of *D. asperoides* roots from different habitats in China (Jin et al., 2020), our study identified some genes highly related to saponin biosynthesis through analyzing differentially proteins and binding transcriptome analysis in three tissues. In the analysis of transcriptome and proteomics, some genes were simultaneously identified, such as IDI (transcript-3950 and transcript-40019), HMGS (transcript-18826), ACAT (transcript-23660 and transcript-24461), and MVD (transcript-25465), etc. To some extent, this indicated that proteomics analysis was in keeping with the results in mRNA level.

### 3.6 Co-expression analysis of triterpenoid saponin contents and biosynthesis-associated transcripts

Co-expression analyses were generally used to exploit biological significance genes (Langfelder and Horvart, 2008). WGCNA is a system biology method for disclosing huge related gene clusters to different ingredients and figure out correlation coefficients between modules and target ingredients. It is convenient to seek out the modules related to triterpenoid saponins in tissues for further identifying critical genes involved in the biosynthesis of saponin. Genes involved in saponin biosynthesis of *D. asperoides* were identified through co-expression analysis and WGCNA. In this study, both saponin and non-saponin components were jointly analyzed to more accurately disclose genes related to triterpenoid saponin biosynthesis. As shown in **Figure 3**, a total of 18,940 transcripts were subdivided into twelve modular clusters based on transcripts expression levels and relative content of compounds, and all modules were inconsistently correlated with different saponins. This was conducted to disclosing the correlation of tissues and triterpenoid saponin contents. Genes with a positive correlation related to a certain saponin identified in modules can be selected for preferred candidate genes for further enzymatic function verification. Based on this, genes associated with a certain type of saponin biosynthesis were screened according to the coefficients (R > 0.5) and *p*-values (*p* < 0.05). For instance, in the MEmagenta module, 137 transcripts were remarkably associated with hederacoside C (R = 0.8, *p* < 0.05) and asperosaponin VI (R = 0.79, *p* < 0.05) composition in specimens, while dipsacoside B (R = -0.55, *p* < 0.05) showed negative correlation comparing with the above transcripts. Transcripts in the MEblue module displayed a highly positive correlation with hederagenin (R = 0.9, *p* < 0.05), while alpha-hederin exhibited a negative correlation. In MEgreen module, dipsacoside B (R = 0.81, *p* < 0.05) was significantly correlated with 344 transcripts. For non-saponin components, 131 transcripts in the MEpurple module were significantly correlated with loganin (R = 0.77, *p* < 0.05). In MEmagenta module, there was a positive relationship between 137 transcripts and sweroside (R = 0.91, *p* < 0.05), and 185 transcripts in the MEpink module were remarkably associated with loganic acid (**Figure 3**). Consequently, 1,256 transcripts were identified in seven modules correlated with target compositions. The red, yellow and bule modules contained saponins-type genes, but the purple, magenta, and pink modules contained non-saponin-type genes. More attention should be paid to red, yellow and bule modules for effectively screening essential genes participated in triterpenoid saponins of biosynthesis pathways in *D. asperoides*.

Different types of genes from modules positively correlated with triterpenoid saponin contents were obtained by WGCNA (**Figure 3**). In MEyellow and MEmagenta, 603 and 137 transcripts were strongly correlated with asperosaponin VI and hederacoside C, respectively. Furthermore, 284 and 343 transcripts were positively correlated with dipsacoside B in MEred and MEgreen modules, respectively. Moreover, 644 transcripts were highly associated with hederagenin in MEblue module. In total, 6 CYPs and 24 UGTs transcripts were identified, which were positively related to triterpenoid saponin contents. Four CYPs (transcript-25629, transcript-16020, transcript-23553, transcript-26545) and three UGTs (transcript-28964, transcript-34905, transcript-22101) were highly associated with dipsacoside B. CYP (transcript-24386) and UGTs (transcript-41, transcript-1640, transcript-2566, transcript-6158, transcript-14975, transcript-24644, transcript-25899 and transcript-27569) were strongly associated with hederagenin. In addition, CYP (transcript-20499) and UGTs (transcript-7971, transcript-8621, transcript-9918, transcript-11311, transcript-11633, transcript-13827, transcript-15957, transcript-17374, transcript-25075, transcript-26318, transcript-27530, transcript-30396) were strongly correlated with asperosaponin VI and hederacoside C (**Figure 3**, **Supplementary Tables 3 and 4**).

**Figure 6:**
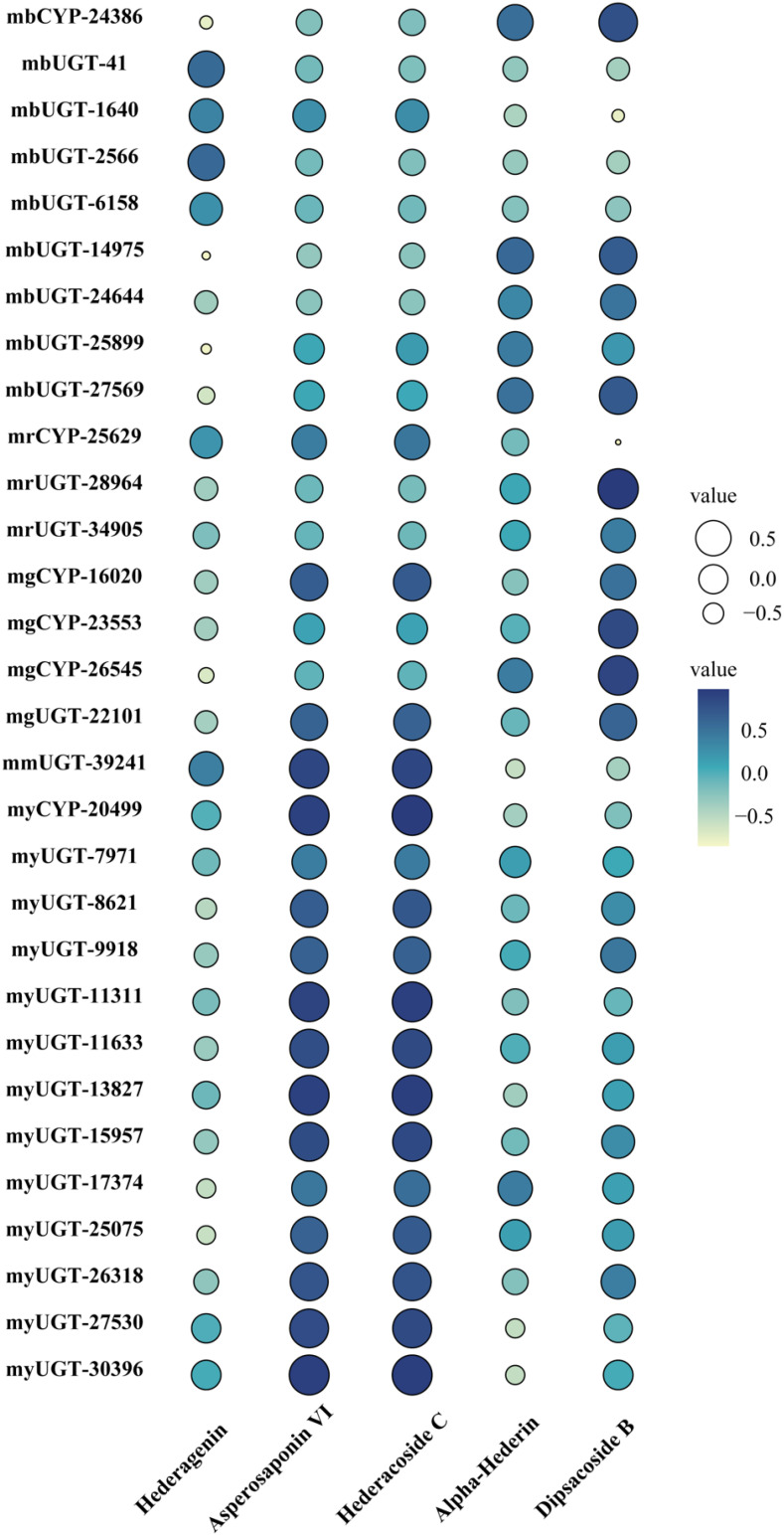
Pearson correlation bubble chart of gene expression patterns and saponin contents in five tissues of *D. asperoides*. mb, MEblue; mr, MEred; mg, MEgreen; mm, MEmagenta; my, MEyellow. The size of circles corresponds to correlation coefficient (R) values, and colors indicate whether a correlation is negative or positive.

CYPs and UGTs play important roles in saponins biosynthesis. It was found that 6 transcripts of CYPs and 24 transcripts of UGTs were highly expressed in five WGCNA modules (**Figure 6**). In **Supplementary Table 4**, it was summarized the correlation between saponins contents and the above genes examined. It is obvious that the significant correlation of seven UGTs (transcript-8621, transcript-11311, transcript-11633, transcript-13827, transcript-15957, transcript-25075 and transcript-26318) and one CYP (transcript-20499) was prominently positively correlated with hederacoside C and asperosaponin VI. Conversely, the above transcripts were inversely associated with hederagenin. Hederagenin can be catalyzed to hederacoside C and asperosaponin VI by UGTs, indicating that these genes could contribute to the biosynthesis of hederacoside C and asperosaponin VI. In addition, the expression of three CYPs (transcript-24386, transcript-23553 and transcript-26545) and two UGTs (transcript-27569 and transcript-28964) were notably correlated with dipsacoside B. As a result, those genes could be potentially related to the biosynthesis of dipsacoside B. Furthermore, there were one CYP (transcript-24386) and two UGTs (transcript-14975 and transcript-27569) were likely involved in the biosynthesis of alpha-hederin. It is worth mentioning that two CYPs (transcript-26545 and transcript-23553) were also identified in proteomics. These results will provide novel insights into understanding the biological functions of target genes in *D. asperoides*.

## 4 Conclusion

In summary, this is the first report on the full-length transcriptome of the medicinal plant *D. asperoides*. The distribution and contents of saponins exhibited tissue-specific dependent patterns in *D. asperoides*. Candidate CYPs, UGTs and other transcripts involved triterpenoid saponins biosynthesis were finally revealed through an integrated analysis strategy of the transcriptome, proteomics, and metabolites in five various tissues of *D. asperoides*, including root, leaf, flower, stem, and fibrous root. Together, these findings will offer novel insights into the molecular level for the control and regulation of saponin biosynthesis in *D. asperoides* and genetic elements for synthetic bioactive natural active compounds *de novo*.

## Data availability statement

The data presented in the study are deposited in the NCBI repository, accession number PRJNA889678, and ProteomeXchange, accession number PXD038580.

## Author contributions

RW and XY were the leading investigators of this research program. RW designed the experiments. JP and CH performed most of the experiments and analyzed the data. WY, TN and XY assisted in experiments and discussed the results. JP and RW wrote the manuscript. All authors contributed to the article and approved the submitted version.
