# Supplementary material for: Full-length transcriptome, proteomics and metabolite analysis reveal candidate genes involved triterpenoid saponin biosynthesis in Dipsacus asperoides
Source: Front Plant Sci. 2023 Feb 10;14:1134352. doi: 10.3389/fpls.2023.1134352 (PMC9950739; doi:10.3389/fpls.2023.1134352)
Supplement: Supplementary file 1 [file DataSheet_1.docx]

Full-length transcriptome, proteomics and metabolite analysis reveal candidate genes involved triterpenoid saponin biosynthesis in *Dipsacus asperoides*

Jie Pan^1,2^, Chaokang Huang^1,2^, Weilin Yao^1,2^, Tengfei Niu^1,2^, Xiaolin Yang^1,2,4^*, Rufeng Wang^1,2,3,4^*

^1^Institute of Chinese Materia Medica, Shanghai University of Traditional Chinese Medicine, Shanghai 201203, P.R. China

^2^The SATCM Key Laboratory for New Resources and Quality Evaluation of Chinese Medicines, Shanghai 201203, P.R. China

^3^The MOE Key Laboratory for Standardization of Chinese Medicines, Shanghai University of Traditional Chinese Medicine, Shanghai 201203, P.R. China.

^4^Shanghai R&D Center for Standardization of Chinese Medicines, Shanghai 201203, P.R. China.

*** Correspondence:**

Rufeng Wang
wrffrw0801@shutcm.edu.cn

Xiaolin Yang

xiaolinysn@126.com

Supplementary Material

# Supplementary Figures and Tables

## Supplementary Tables

**Supplementary Table 1.** Overview of sequence data quality obtained from Illumina sequencing.

| **Sample** | **Raw reads** | **Clean reads** | **Clean bases** | **Error (%)** | **Q20 (%)** | **Q30 (%)** | **GC (%)** |
| --- | --- | --- | --- | --- | --- | --- | --- |
| root-1 | 50263032 | 49653226 | 6.63G | 0.03 | 96.8 | 91.37 | 43.51 |
| root-2 | 49217704 | 48602848 | 6.49G | 0.03 | 96.66 | 91.09 | 43.5 |
| root-3 | 55265868 | 54616634 | 7.28G | 0.03 | 96.79 | 91.34 | 43.59 |
| flower-1 | 57023694 | 54439558 | 7.19G | 0.03 | 96.77 | 91.35 | 43.21 |
| flower-2 | 51180284 | 48641816 | 6.46G | 0.03 | 96.76 | 91.32 | 43.35 |
| flower-3 | 54127024 | 51514784 | 6.82G | 0.03 | 96.94 | 91.69 | 43.35 |
| leaf-1 | 46407960 | 45770944 | 5.97G | 0.03 | 96.67 | 91.12 | 42.4 |
| leaf-2 | 49397392 | 48657018 | 6.34G | 0.03 | 97.16 | 92.13 | 42.24 |
| leaf-3 | 48772436 | 47936442 | 6.24G | 0.03 | 97.11 | 92.07 | 42.22 |
| fibrous root-1 | 44741948 | 41454834 | 5.44G | 0.03 | 96.78 | 91.34 | 42.35 |
| fibrous root-2 | 51121362 | 47850146 | 6.3G | 0.03 | 96.68 | 91.12 | 42.66 |
| fibrous root-3 | 50936294 | 47660344 | 6.26G | 0.03 | 96.79 | 91.32 | 42.4 |
| stem-1 | 53614856 | 52543320 | 6.89G | 0.03 | 97.25 | 92.33 | 43.51 |
| stem-2 | 49585438 | 48461022 | 6.31G | 0.03 | 97.11 | 92.09 | 43.44 |
| stem-3 | 53596020 | 52284014 | 6.83G | 0.03 | 97.25 | 92.36 | 43.49 |
| Total | 765251312 | 740086950 | 97.54G |  |  |  |  |

**Supplementary Table 5.** The up-regulated and down-regulated quantitative proteins in different tissue of *D. asperoides* (*p*-values ≤ 0.05 and fold change ≥ 2).

| Comparisons | Significantly changing in abundance | | |
| --- | --- | --- | --- |
|  | Up-regulated | Down-regulated | All |
| Proot -VS- Pleaf | 40 | 62 | 102 |
| Proot -VS-Pflower | 38 | 94 | 132 |
| Pleaf -VS- Pflower | 333 | 407 | 740 |

## Supplementary Figures


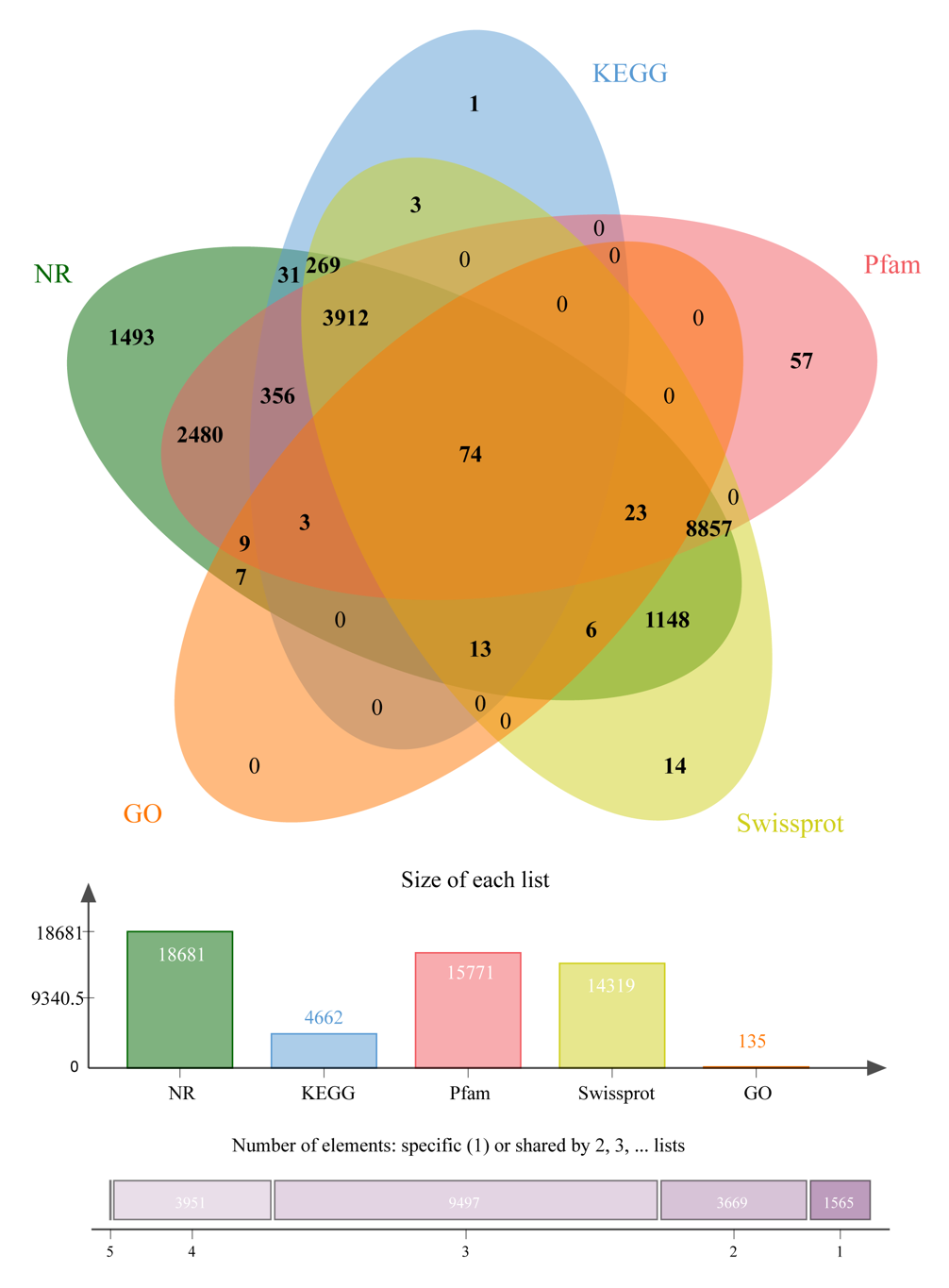


**Supplementary Figure 1.** Gene function annotation results.


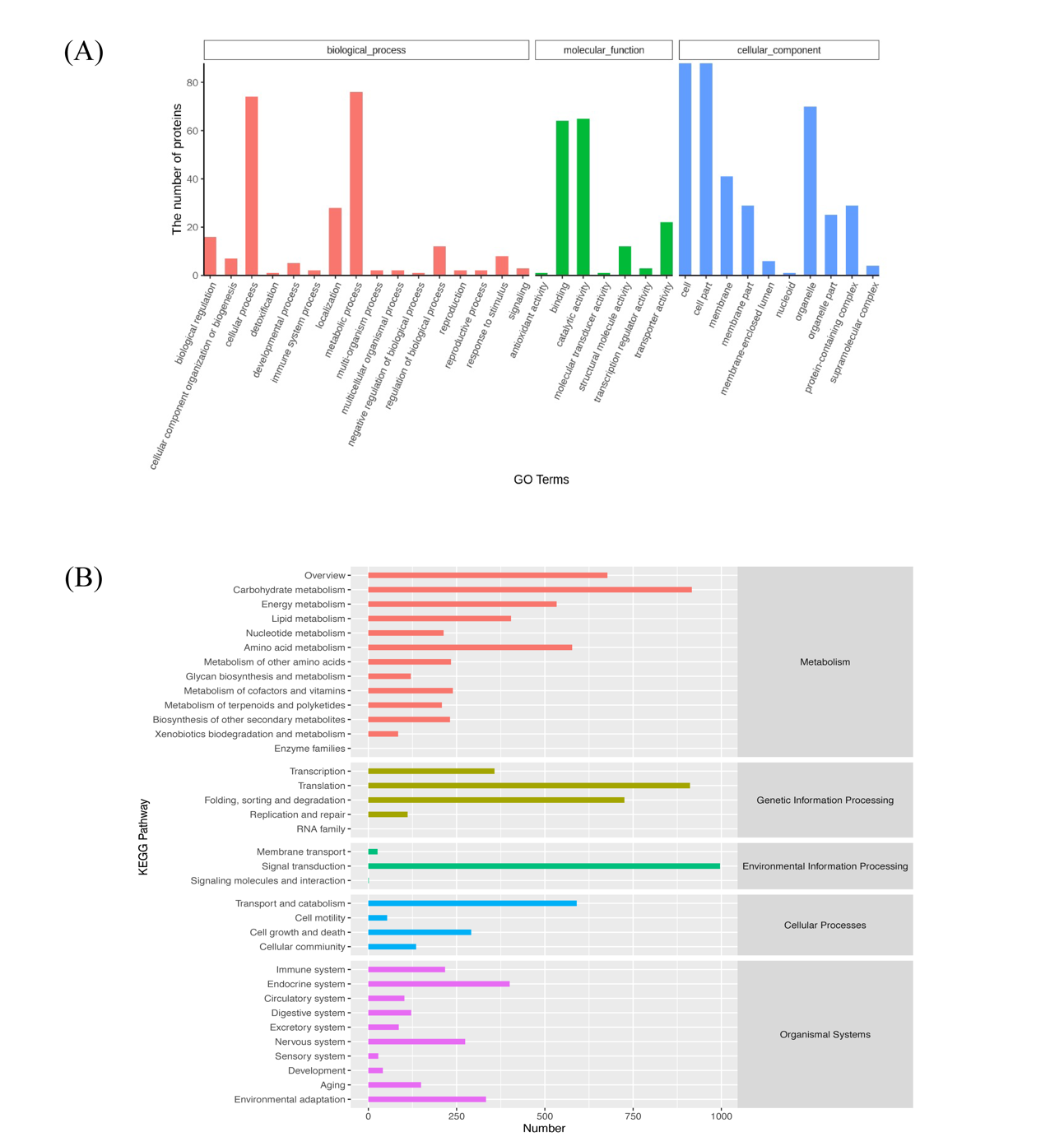


**Supplementary Figure 2.** GO and KEGG Gene function annotation results based on the transcriptome. (A) GO annotated for *D. asperoides* tissues (root, leaf, flower, stem, fibrous root) at the levels of biological processe (BP), cellular component (CC), and molecular function (MF). (B) KEGG annotation of *D. asperoides* tissues (root, leaf, flower, stem, fibrous root).


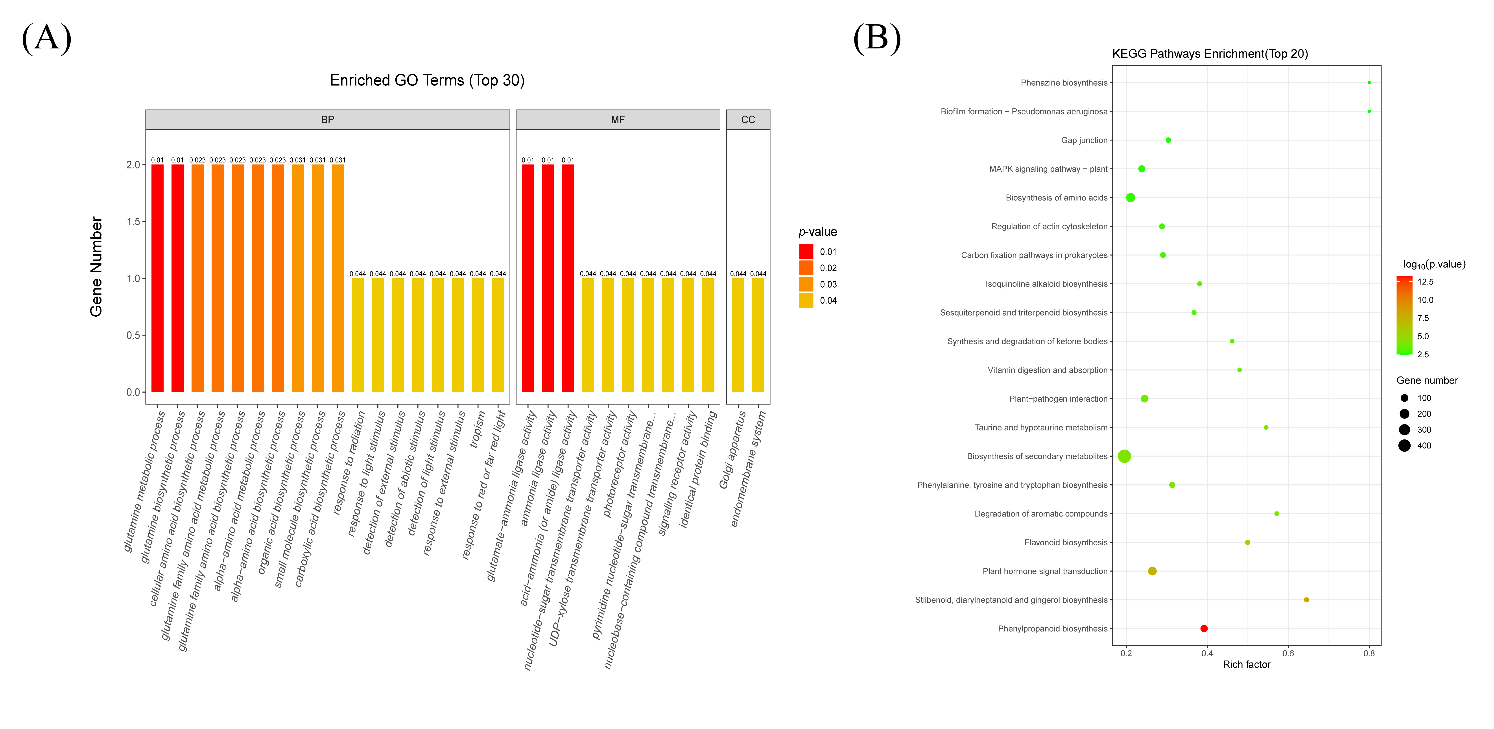


**Supplementary Figure 3.** GO and KEGG enrichment analysis results based on the differentially upregulated genes in root and flower tissues from transcriptome. (A) GO annotated for *D. asperoides* tissues (root and flower) at the levels of biological processe (BP), cellular component (CC), and molecular function (MF). (B) KEGG enrichment analysis of *D. asperoides* tissues (root and flower).


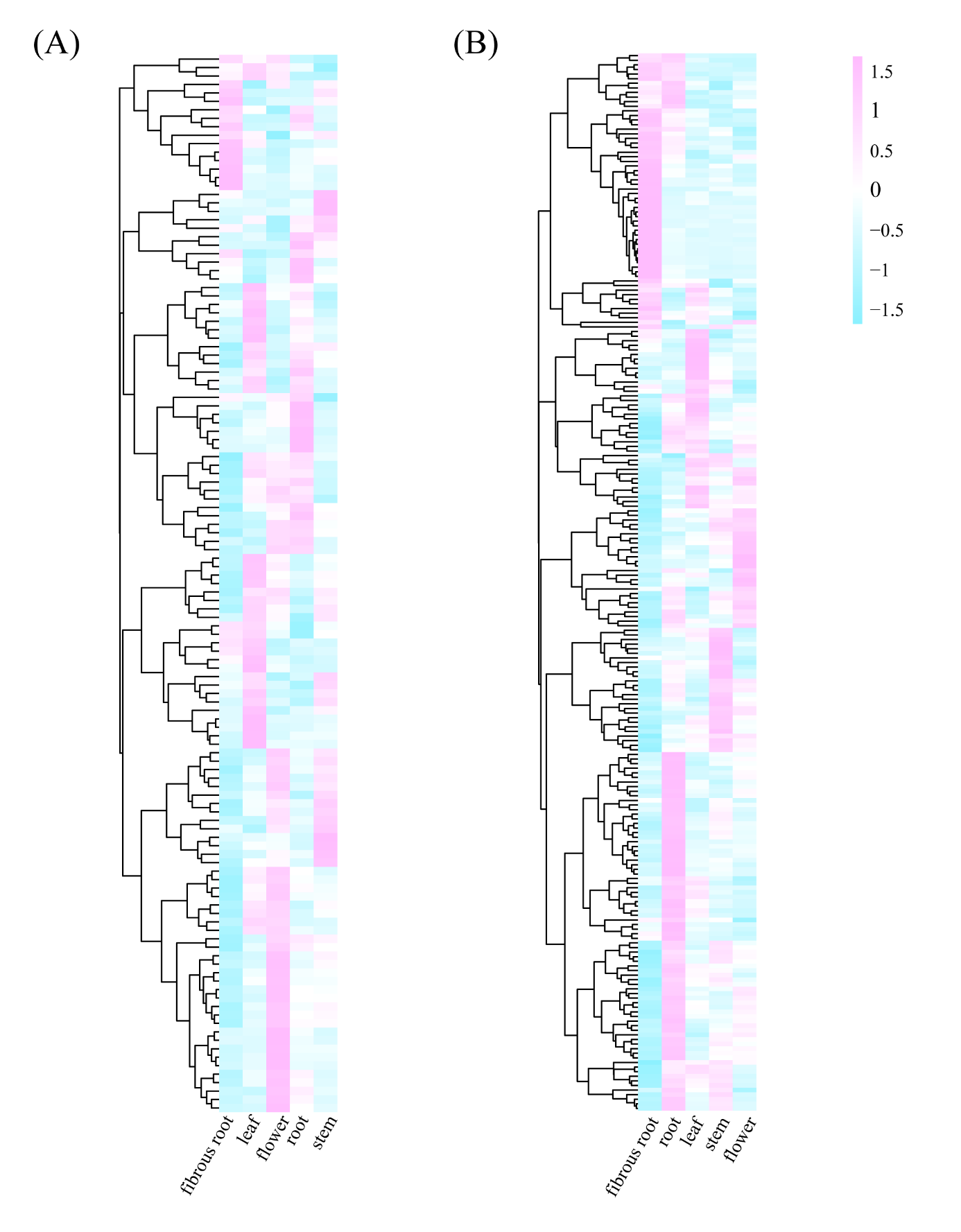


**Supplementary Figure 4.** CYP and UGT gene expression involved in saponins biosynthesis of *D. asperoides*. (A) Heatmap of the candidate CYP gene expression patterns in five tissues from *D. asperoides*. (B) Heatmap of the candidate UGT gene expression patterns in five tissues from *D. asperoides*. (All of the transcript codes/names kept in Supplementary Table 3).


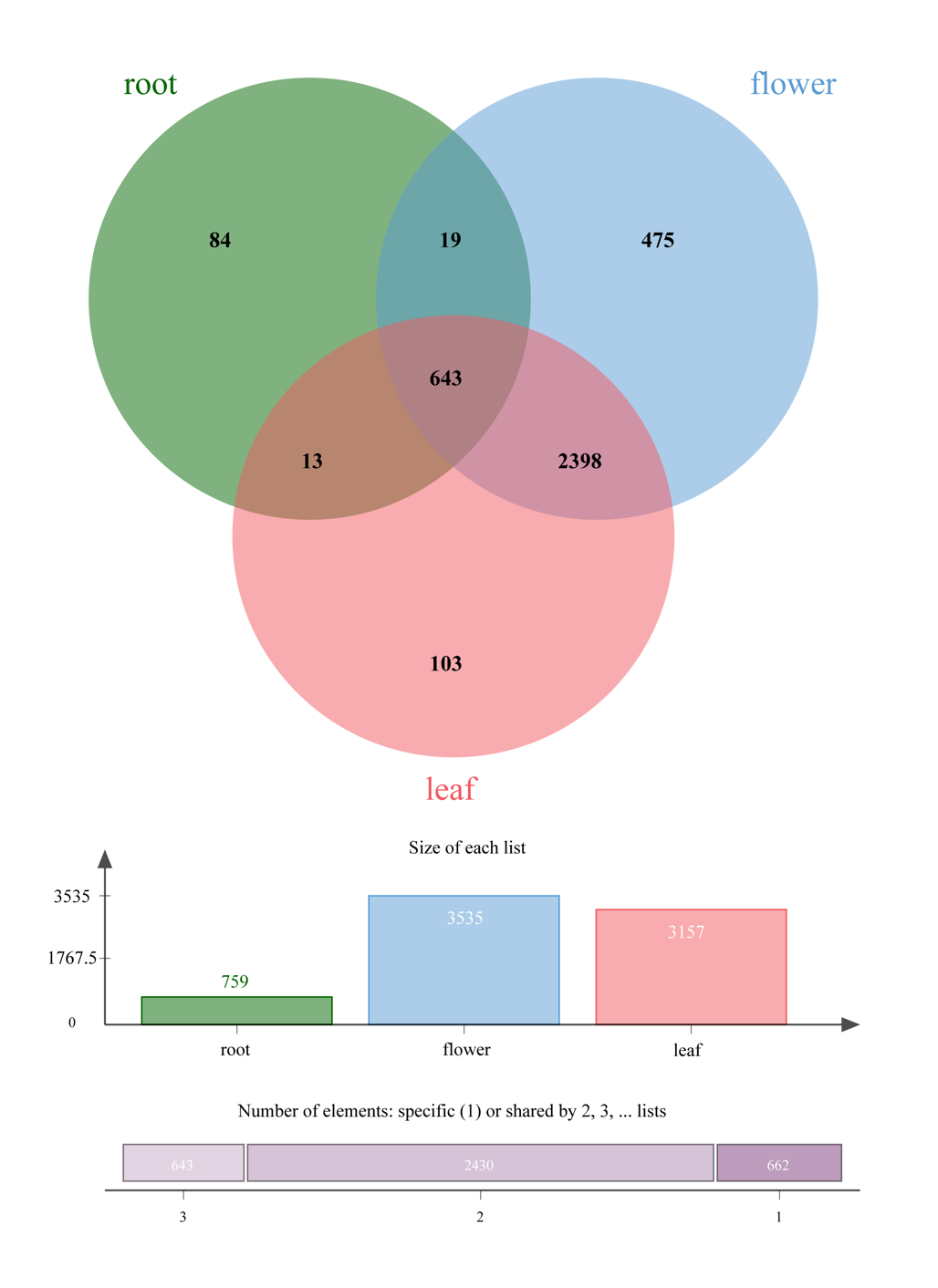


**Supplementary Figure 5.** A Venn diagram comparing proteins expression between different tissues (root, flower, leaf) from *D. asperoides*.


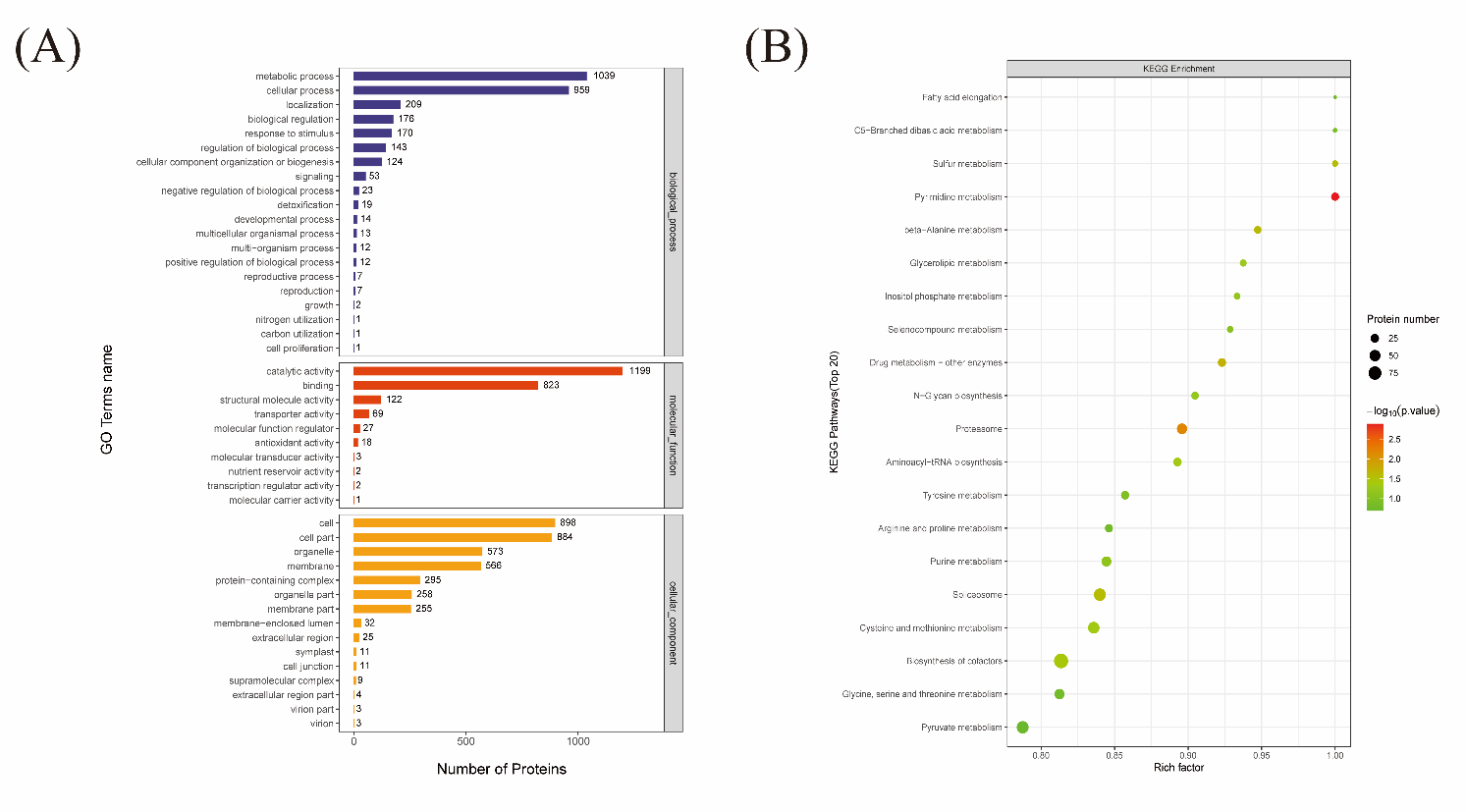


**Supplementary Figure 6.** GO and KEGG enrichment analysis results based on the different proteins in root and flower tissues from proteomics. (A) GO annotated for *D. asperoides* tissues (root and flower) at the levels of biological processe (BP), cellular component (CC), and molecular function (MF). (B) KEGG enrichment analysis of *D. asperoides* tissues (root and flower).


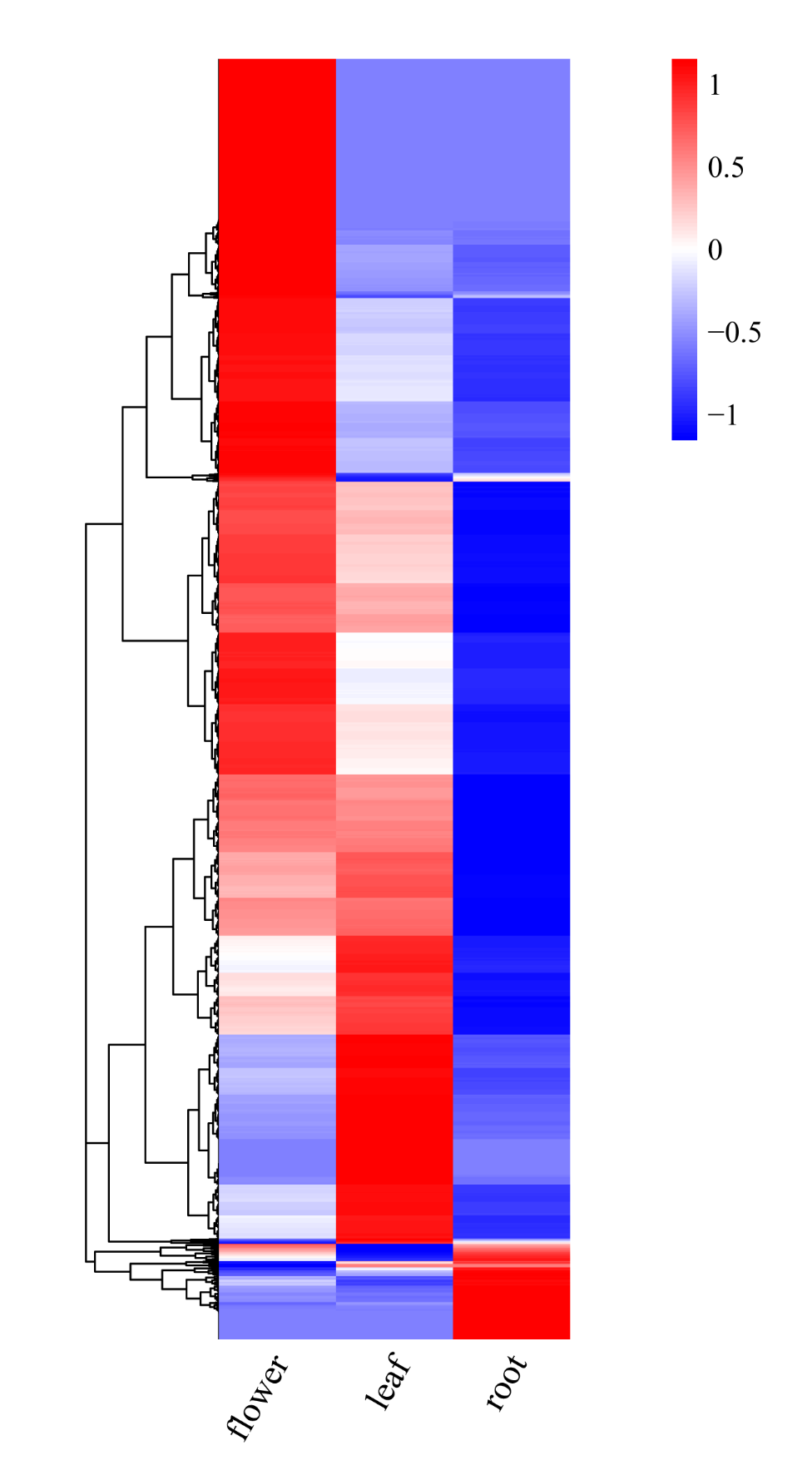


**Supplementary Figure 7.** Heatmap of all proteins expression level in three tissues from *D. asperoides*. (All of the transcript codes/names kept in Supplementary Table 6).
